# 2-Aminoethoxydiphenylborate (2-APB) inhibits release of phosphatidylserine-exposing extracellular vesicles from platelets

**DOI:** 10.1038/s41420-020-0244-9

**Published:** 2020-03-02

**Authors:** Hao Wei, Jessica E. Davies, Matthew T. Harper

**Affiliations:** Department of Pharmacology, University of Cambridge, Cambridge, UK

**Keywords:** Mechanism of action, Drug development

## Abstract

Activated, procoagulant platelets shed phosphatidylserine (PS)-exposing extracellular vesicles (EVs) from their surface in a Ca^2+^- and calpain-dependent manner. These PS-exposing EVs are prothrombotic and proinflammatory and are found at elevated levels in many cardiovascular and metabolic diseases. How PS-exposing EVs are shed is not fully understood. A clearer understanding of this process may aid the development of drugs to selectively block their release. In this study we report that 2-aminoethoxydiphenylborate (2-APB) significantly inhibits the release of PS-exposing EVs from platelets stimulated with the Ca^2+^ ionophore, A23187, or the pore-forming toxin, streptolysin-O. Two analogues of 2-APB, diphenylboronic anhydride (DPBA) and 3-(diphenylphosphino)-1-propylamine (DP3A), inhibited PS-exposing EV release with similar potency. Although 2-APB and DPBA weakly inhibited platelet PS exposure and calpain activity, this was not seen with DP3A despite inhibiting PS-exposing EV release. These data suggest that there is a further target of 2-APB, independent of cytosolic Ca^2+^ signalling, PS exposure and calpain activity, that is required for PS-exposing EV release. DP3A is likely to inhibit the same target, without these other effects. Identifying the target of 2-APB, DPBA and DP3A may provide a new way to inhibit PS-exposing EV release from activated platelets and inhibit their contribution to thrombosis and inflammation.

## Introduction

Platelets are the main cellular component of haemostasis. They adhere, activate and aggregate at sites of vascular injury, forming a haemostatic plug that prevents blood loss. However, platelet activation on a ruptured coronary atherosclerotic plaque is the main event in coronary thrombosis, leading to unstable angina or myocardial infarction^1^. Anti-platelet drugs are therefore used to prevent coronary thrombosis in at-risk patients^2,3^.

During platelet activation, procoagulant platelets expose phosphatidylserine (PS) in the outer leaflet of their plasma membrane. PS is normally restricted to the inner leaflet by an aminophospholipid translocase (‘flippase’). A high, sustained increased in cytosolic Ca^2+^ concentration ([Ca^2+^]_cyt_) leads to inhibition of the flippase and activation of a non-selective phospholipid scramblase, TMEM16F^4–6^. The result is net movement of PS to the outer leaflet. PS forms a procoagulant surface for the tenase and prothrombinase coagulation complexes, increasing thrombin generation^7–9^.

Procoagulant platelets also release PS-exposing extracellular vesicles (EVs). These are prothrombotic and proinflammatory^10,11^. The levels of PS-exposing EVs are increased in many cardiovascular and metabolic disorders^12–17^, and platelets are the major source of circulating PS-exposing EVs^11,18^. The pathological role of PS-exposing EVs, and their elevation in a range of diseases, makes them an attractive therapeutic target.

How PS-exposing EVs are shed from the plasma membrane is not fully understood. A clearer understanding of this process may aid the development of drugs to selectively block their release. PS exposure itself may be important, as they are not shed from platelets from *Tmem16f*^−*/−*^ mice or from patients with Scott Syndrome, a very rare bleeding disorder caused by lack of functional TMEM16F^5,19^. In addition, high [Ca^2+^]_cyt_ in procoagulant platelets activates the Ca^2+^-dependent protease, calpain^20,21^. Calpain-dependent cleavage of cytoskeletal proteins is also necessary. However, little is known of the mechanism beyond these events. Indeed, it is not clear whether any events other than PS exposure and calpain activity are required downstream of increased [Ca^2+^]_cyt_.

Platelets can be effectively stimulated to shed PS-exposing EVs by Ca^2+^ ionophores, such as A23187, triggering a high, sustained increase in [Ca^2+^]_cyt_. Although not a physiological stimulus, by bypassing platelet receptors and their proximal signalling cascades it can be used to understand the downstream mechanisms of EV release. PS-exposing EVs can be distinguished by their size and their capacity to bind annexin V (AnV). During preliminary experiments, we were surprised to find that 2-aminoethoxydiphenylborate (2-APB) significantly inhibited A23187-induced annexin V-positive (AnV^+^) EV release. This was surprising since 2-APB is a cell-permeable modulator of numerous ion channels, particularly Ca^2+^ channels, yet we expected the use of a Ca^2+^ ionophore to bypass any contribution of plasma membrane or intracellular Ca^2+^ channels. Therefore, in this study, we investigated the mechanism of how 2-APB inhibits the release of AnV^+^ EVs from platelets. We find that the effect of 2-APB does not depend on inhibition of Ca^2+^ signalling, PS exposure or calpain activity. These results indicate that events in addition to PS and calpain activation are required for release of PS-exposing EVs and suggest the existence of other potential targets to inhibit this important pathological process.

## Results

### 2-APB inhibits AnV^+^ EV release induced by the Ca^2+^ ionophore, A23187

**Fig. 1:**
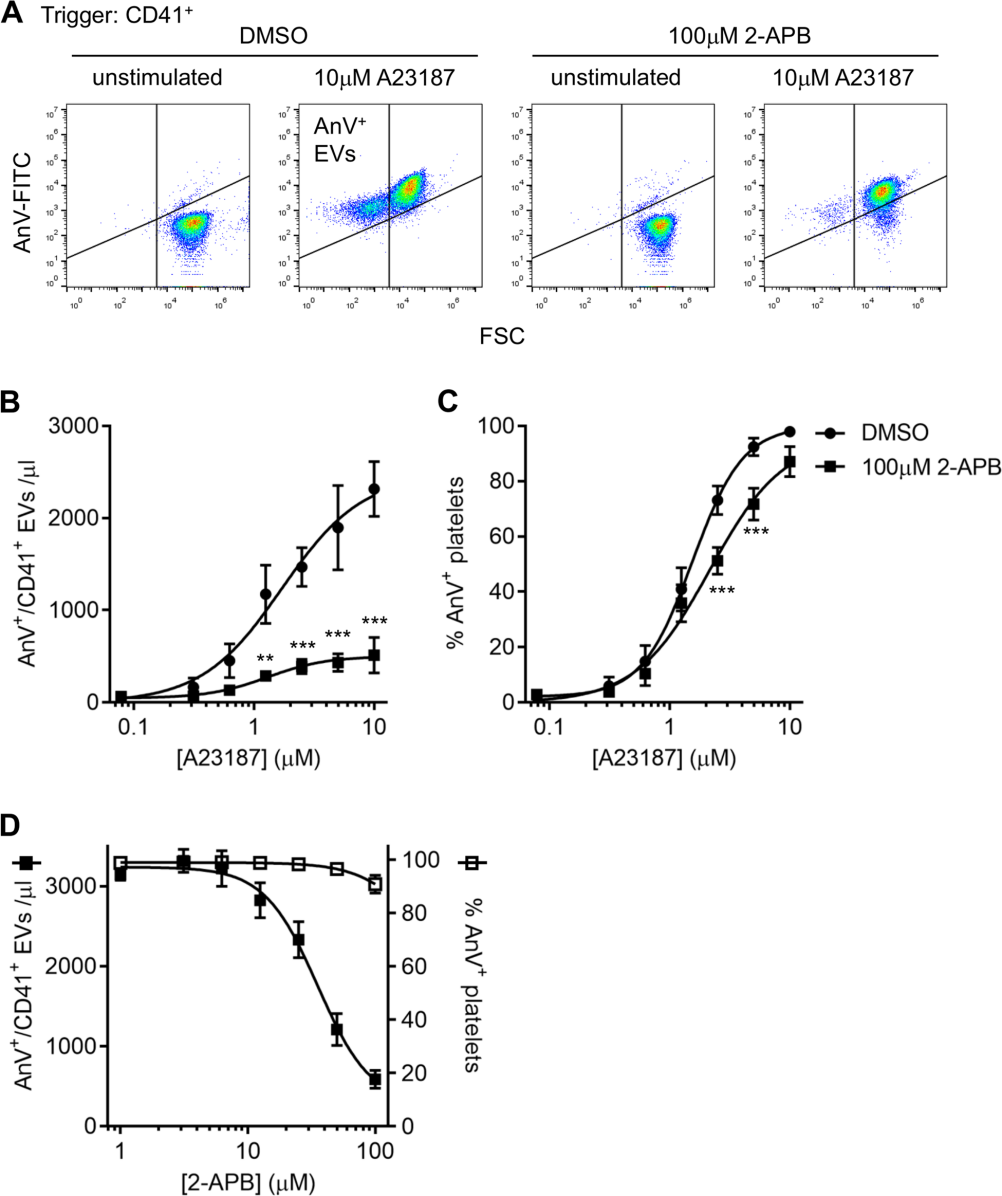
2-APB inhibits release of PS-exposing (AnV^+^) extracellular vesicles (EVs). **a** Washed platelets were treated with 2-APB (100 μM; 30 min) or the solvent, DMSO, as control, then stimulated with the indicated concentration of A23187 for 10 min, after which samples were stained with anti-CD41a-PerCP-Cy7, and annexin V-FITC to detect PS exposure. PerCP-Cy7 fluorescence was used to trigger acquisition of CD41^+^ events. The panels show density plots of events from low density (blue) to high density (red) of forward scatter (FSC) and FITC fluorescence. Unstimulated platelets have high FSC-A and low annexin V-FITC binding (LR). Stimulation with A23187 triggered PS exposure in platelets and release of PS-exposing (AnV^+^) EVs. The vertical line separating left and right was defined by the FSC of 1 µm silica beads. The density plots are representative of data from five different donors. **b**, **c** Platelets were stimulated with the indicated concentration range of A23187. Mean data (±s.e.m.; *n* = 5) are shown for AnV^+^ EVs (**b**) and the percentage of platelets (>1 µm) binding AnV. Concentration–response curves were fitted with a four-parameter logistic equation. Data were further analysed by two-way RM-ANOVA with Sidak’s post-test. **p < 0.01; ***p < 0.001 (*n* = 5). **d** Platelets were treated with a range of concentrations of 2-APB prior to stimulation with 10 µM A23187 (*n* = 5).

In order to better understand the processes by which platelets release PS-exposing EVs, platelets were stimulated with the Ca^2+^ ionophore, A23187. This triggers rapid Ca^2+^ entry, resulting in PS exposure and release of PS-exposing EVs. PS exposure was detected using annexin V (AnV) (Fig. 1a). AnV^+^ platelets and AnV^+^ EVs were detected by flow cytometry. As we have previously discussed, this approach is likely to preferentially detect the largest AnV^+^ EVs and to underestimate the total number of EVs released^22^. However, it is still a rapid and effective means of assessing AnV^+^ EV release.

Pre-treatment with 2-APB (100 µM) significantly inhibited AnV^+^ EV release compared to platelets treated with the vehicle (DMSO) at all concentrations of A23187 tested. In contrast, the percentage of AnV^+^ platelets was only weakly affected (Fig. 1a–c). When 10 µM A23187 was used, 2-APB inhibited AnV^+^ EV release with a pIC_50_ of 4.45 ± 0.09 (IC_50_ approximately 35 µM; *n* = 5), with no corresponding effect on platelet AnV^+^ binding (Fig. 1d).

To assess the reversibility of the inhibition, platelets were treated with 2-APB or DMSO then washed by centrifugation. The procedure of washing platelets by centrifugation resulted in slightly greater sensitivity to A23187 (pEC_50_ of 5.77 ± 0.20 for AnV^+^ EV release prior to washing; pEC_50_ of 6.16 ± 0.12 after washing). 2-APB-treated platelets released significantly fewer AnV^+^ EVs when stimulated with A23187. Notably, there was now no difference in platelet AnV binding between 2-APB-treated and DMSO-treated platelets at any concentration of A23187 (Supplementary Fig. 1).

Together, these data indicate that 2-APB inhibits A23187-induced AnV^+^ EV release. This action not related to the weak inhibition of platelet PS exposure.

### Structural analogues of 2-APB show differing effects on AnV^+^ EV release

2-APB has many reported targets. To further characterise its effects, we used a small panel of related, commercially available compounds (Supplementary Fig. 2). The effects of these analogues on some 2-APB targets has been previously described (Supplementary Table 1).

**Fig. 2:**
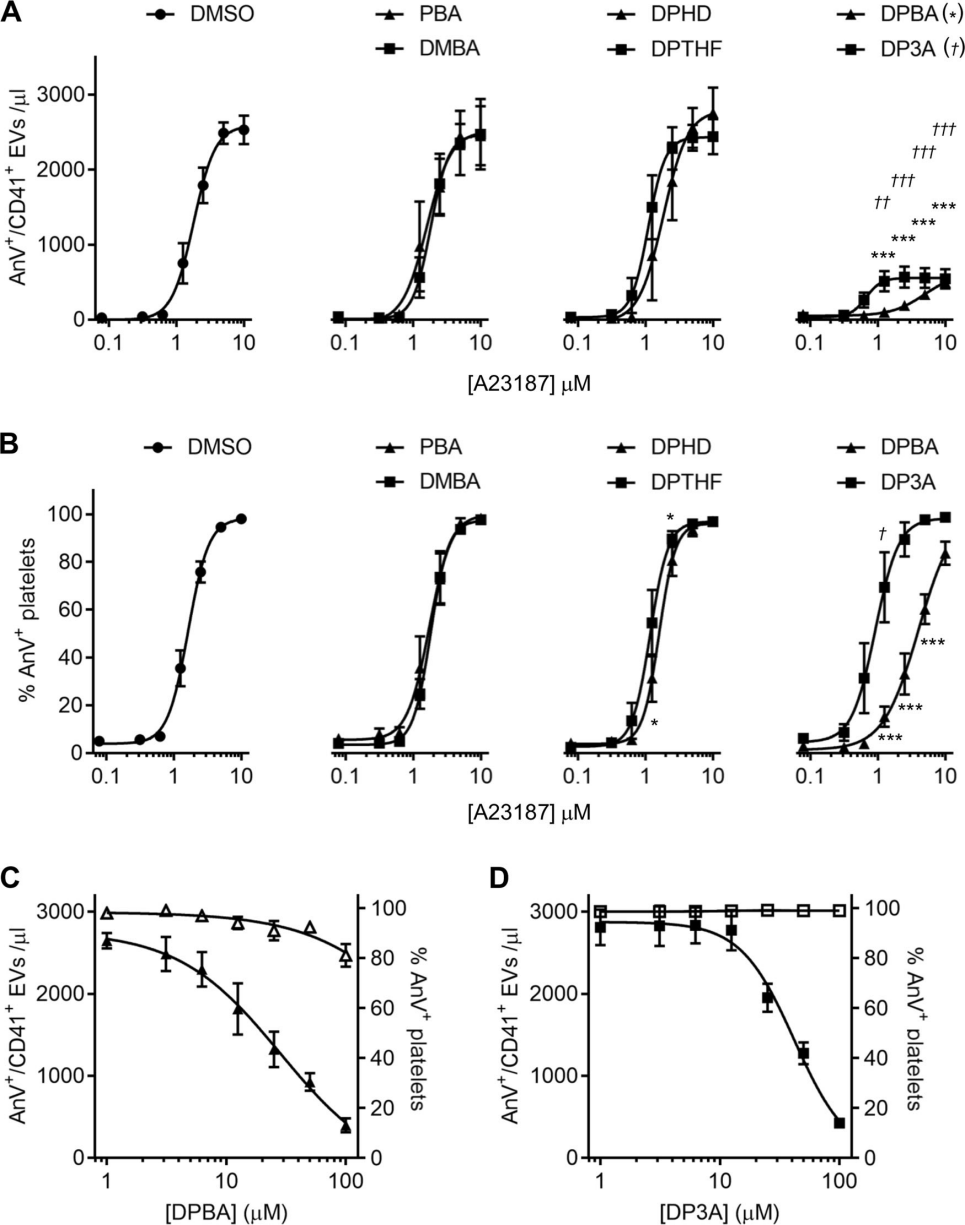
Structural analogues of 2-APB show differing effects on AnV^+^ EV release. **a**, **b** Platelets were treated with the indicated analogue (100 µM; 30 min), or DMSO as control, then stimulated with a concentration range of A23187. AnV^+^ EV release and platelet AnV binding were determined as described in Fig. 1. Concentration–response curves were fitted with a four-parameter logistic equation. Data were further analysed by two-way RM-ANOVA with Sidak’s post-test. *p < 0.05; **p < 0.01; ***p < 0.001 for comparison with DMSO-treated platelets. For clarity, in the right-hand panel, statistical significance for DP3A is marked with † (^††^*p* < 0.01; ^†††^*p* < 0.001) but also indicates comparison with DMSO-treated platelets. **c**, **d** Platelets were treated with a concentration range of DPBA (**c**) or DP3A (**d**) prior to stimulation with A23187 (10 µM).

Some analogues had no effect on A23187-induced AnV^+^ EV release (Fig. 2). Phenylborinic acid (PBA) has the boron atom attached to only one phenyl group and may be too simple a fragment to effectively inhibit AnV^+^ EV release. Since di-PBA is unstable^23^, we used the more stable analogue, dimesitylborinic acid (DMBA), which has two phenyl rings replaced by mesityl groups. This was also ineffective at 100 µM. It may be that analogues with a terminal B–OH are ineffective and that a B–O core is required.

Two analogues that lack the boron atom were also ineffective: 2,2-diphenyltetrahydrofuran (DPTHF), which has a five-membered ring containing an oxygen atom, similar to the ring form of 2-APB, but lacks a boron or nitrogen atom, and diphenhydramine (DPHD), which is similar to the linear form of 2-APB, but lacks the boron atom and has two methyl groups attached to the secondary amine.

In contrast, diphenylboronic anhydride (DPBA) and 3-(diphenylphosphino)-1-propylamine (DP3A) both inhibited AnV^+^ EV release. Both were of similar potency to 2-APB. The pIC_50_ for DPBA was 4.55 ± 0.32 (IC_50_ approximately 28 µM; *n* = 5). The pIC_50_ for DP3A was 4.38 ± 0.14 (IC_50_ approximately 42 µM; *n* = 5). The effectiveness of DPBA and DP3A indicates that neither the amine group nor the boron atom is necessary for inhibition. In addition, DPBA partially reduced the percentage of AnV^+^ platelets, whereas DP3A did not, suggesting that the boron atom may be important for this weak inhibition.

### The inhibitory effect of 2-APB is not through inhibition of Ca^2+^ signalling

**Fig. 3:**
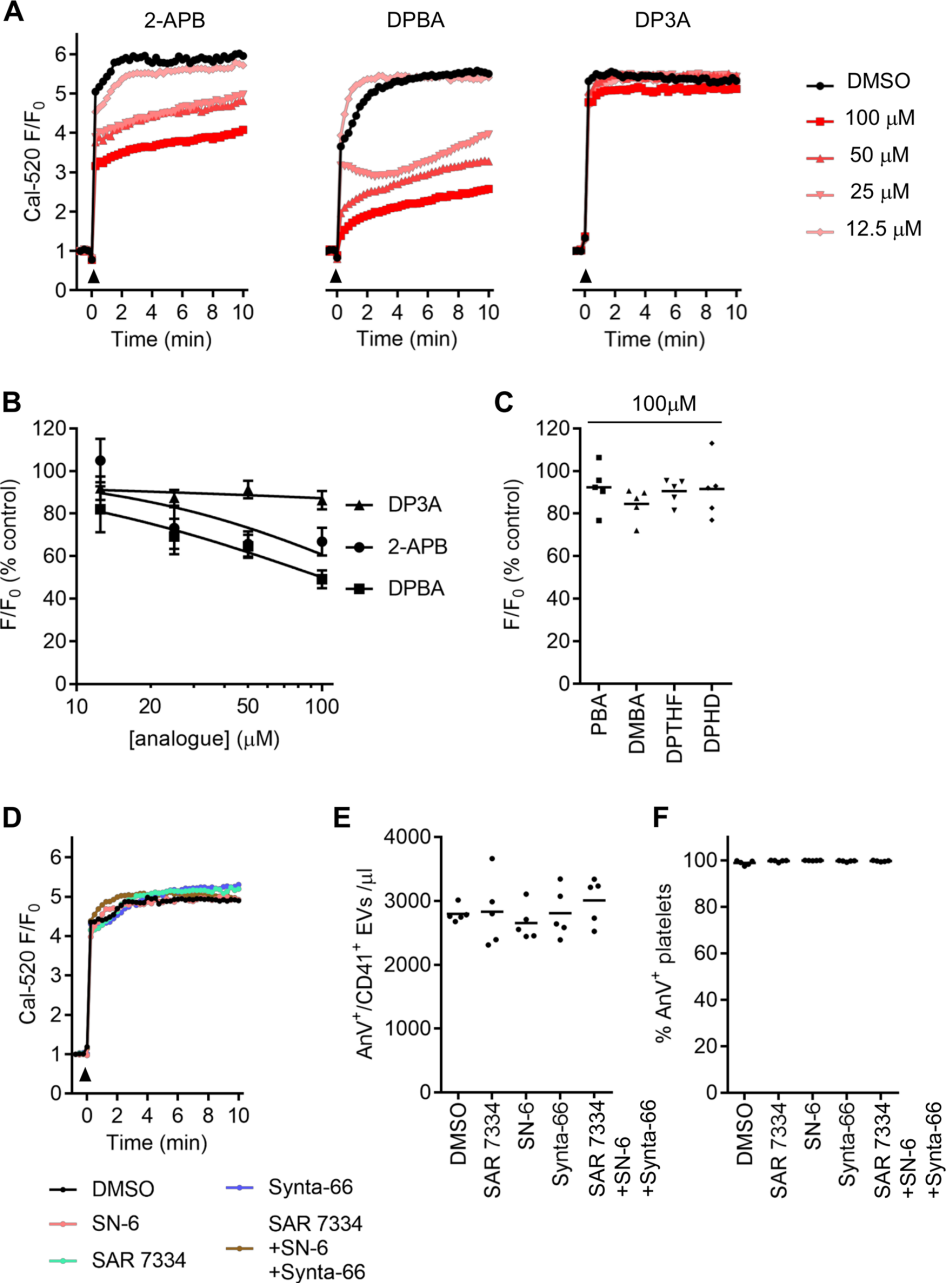
2-APB, DPBA and DP3A reduce Cal-520 fluorescence. **a** Representative traces of Cal-520-loaded platelets treated as indicated then stimulated with A23187 (10 µM; black arrowhead), expressed as Cal-520 fluorescence normalised to the fluorescence prior to stimulation (*F*/*F*_0_). **b** Mean (±s.e.m.; *n* = 5) of maximum *F*/*F*_0_ in each condition, as a percentage of DMSO-treated platelets. **c** Maximum *F*/*F*_0_ for the inactive analogues (line indicated mean). **d** Cal-520-loaded platelets were treated with SAR-7334 (1 µM), SN-6 (50 µM) and Synta-66 (10 µM), individually or combined, as indicated, before stimulation with A23187 (10 µM; black arrowhead). The traces are representative of five independent experiments. **e**, **f** AnV^+^ EV release and platelet AnV binding in samples treated with these inhibitors. No statistically significant difference was observed compared to platelets treated with the solvent, DMSO (in each, one-way RM-ANOVA; *n* = 5).

2-APB is known to modulate a variety of plasma membrane channels that are expressed in platelets, including Orai1, TRPC6 and Ca^2+^-activated K^+^ channels^24–28^. To determine whether any of these targets were involved in the inhibitor effect of 2-APB, Cal-520-loaded platelets were used to monitor the effect of A23187 and 2-APB on cytosolic Ca^2+^ signalling. A23187 induced a rapid increase in fluorescence (Fig. 3a). Surprisingly, 2-APB inhibited this increase in fluorescence in a concentration-dependent manner (Fig. 3a, b). Although DPBA also inhibited the Cal-520 fluorescence in a concentration-dependent manner, DP3A had no effect (Fig. 3a, b). This is important as DP3A inhibited AnV^+^ EV release, suggesting that there is no direct link between the inhibition of Cal-520 fluorescence and inhibition of AnV^+^ EV release. The analogues that did not affect AnV^+^ EV release (PBA, DPTHF, DPHD, and DMBA) had no effect on the A23187-induced increase in fluorescence (Fig. 3c).

Inhibition of Orai1 with Synta-66 (10 μM), TRPC6 with SAR-7334 (1 μM) or the Na^+^–Ca^2+^ exchanger (NCX) with SN-6 (50 μM), singly or combined, had no effect on the A23187-induced increase in Cal-520 fluorescence, AnV^+^ EV release or platelet AnV binding (Fig. 3d–f).

**Fig. 4:**
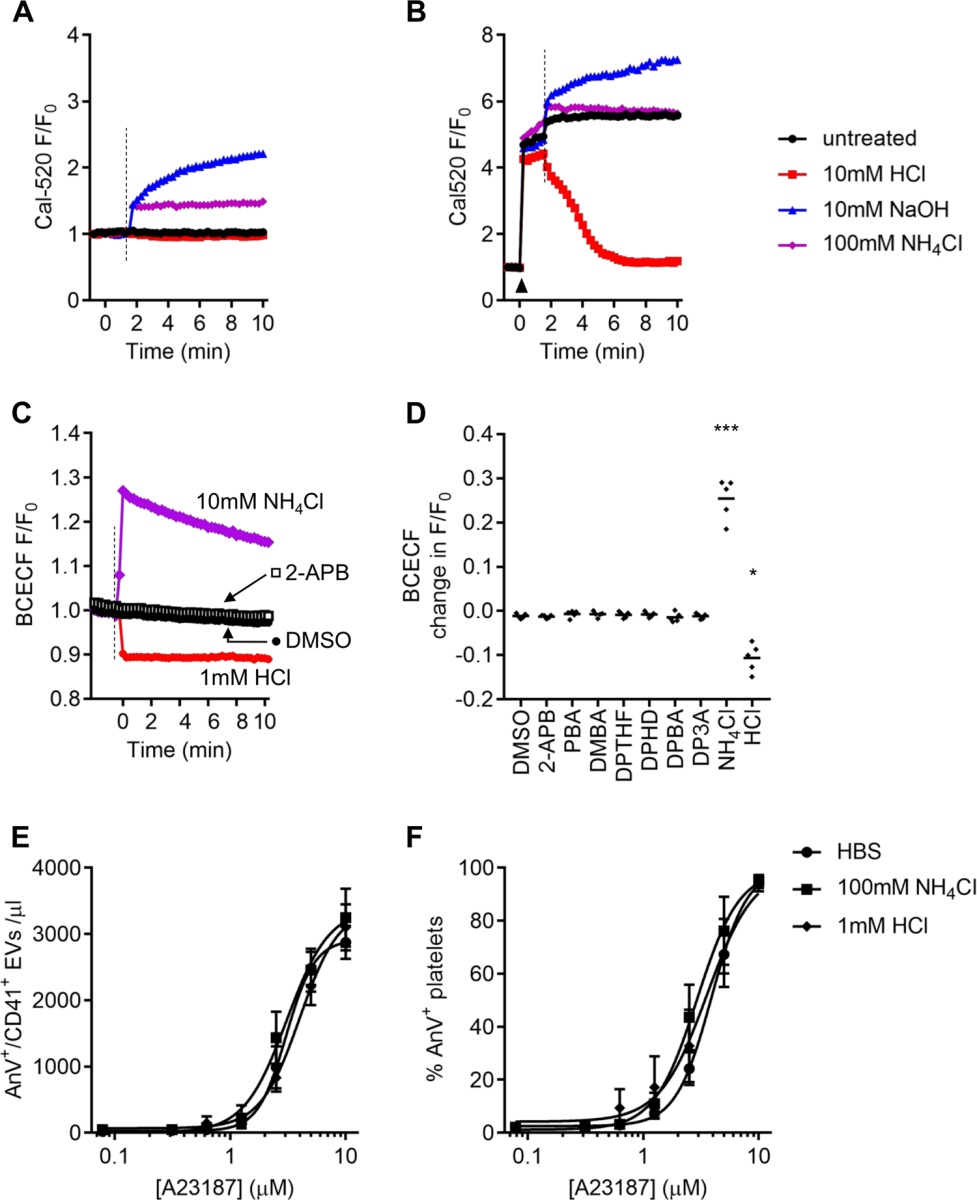
2-APB does not inhibit AnV^+^ EV release by altering cytosolic pH. **a** Cal-520-loaded platelets were treated with HCl (10 mM), NaOH (10 mM) or NH_4_Cl (100 mM) at the point indicated by the dashed line. **b** Cal-520-loaded platelets were stimulated with A23187 (10 µM; black arrowhead) followed by HCl, NaOH or NH_4_Cl (dashed line). The traces are representative of five independent experiments. **c** Platelets were loaded with the pH-sensitive dye, BCECF. HCl, NH_4_Cl, 2-APB or its vehicle, DMSO, were added at the dashed line. **d** Quantification of the change in BCECF fluorescence within 1 min of addition of the compounds indicated. The line shows the mean. Data with each treatment were compared to DMSO-treated platelets by one-way RM-ANOVA, with Dunnett’s post-test. *p < 0.05; ***p < 0.001. **e**, **f** Platelets were treated with NH_4_Cl or HCl prior to stimulation with A23187. Data are mean ± s.e.m. (*n* = 5). No statistically significant differences were observed compared to platelets treated with the solvent, HBS (two-way RM-ANOVA).

Since it was surprising that 2-APB affected A23187-induced cytosolic Ca^2+^ signalling, we investigated whether the effect on Cal-520 fluorescence might be an artefact owing to a change in cytosolic pH. Addition of NH_4_Cl to unstimulated Cal-520-loaded platelets, which is expected to result in rapid intracellular alkalinisation, gave a rapid increase in fluorescence. Similarly, addition of extracellular NaOH also increased the fluorescence. Extracellular HCl had little effect on the Cal-520 fluorescence in unstimulated platelets (Fig. 4a). Although A23187 rapidly increased Cal-520 fluorescence to a plateau, which is as expected as the dye is likely to be saturated with Ca^2+^ under these conditions, this fluorescence could be further increased by extracellular NH_4_Cl or NaOH. It could also be decreased by extracellular HCl, suggesting that H^+^ enters stimulated platelets (Fig. 4b). Together, these data show that Cal-520 fluorescence is highly sensitive to pH.

To determine whether 2-APB affects cytosolic pH, platelets were loaded with the pH-sensitive fluorescent dye, BCECF. NH_4_Cl rapidly increased BCECF fluorescence, indicating cytosolic alkalinisation, which slowly reversed (Fig. 4c). HCl rapidly decreased BCECF fluorescence, indicating cytosolic acidification (Fig. 4c). However, neither 2-APB, nor any of the analogues tested, significantly affected BCECF fluorescence (Fig. 4c, d). This suggests that 2-APB does not affect cytosolic pH. Moreover, the effect of 2-APB and DPBA on Cal-520 fluorescence is not an artefact of decreased cytosolic pH. Furthermore, altering cytosolic pH with NH_4_Cl or HCl did not significantly affect A23187-induced AnV^+^ EV release or platelet AnV binding (Fig. 4e, f). These data indicate that a change in cytosolic pH does not account for the inhibitory effect of 2-APB.

2-APB is also reported to block Ca^2+^-activated K^+^ channels^28^, which may be required for PS-exposing EV release^29^. However, neither quinine (300 μM) nor tetraethylammonium (TEA; 30 mM), two non-selective blockers of Ca^2+^-activated K^+^ channels, inhibited AnV^+^ EV release or platelet AnV binding (Supplementary Fig. 3).

### 2-APB, DBPA and DP3A inhibit AnV^+^ EV release from permeabilised platelets

**Fig. 5:**
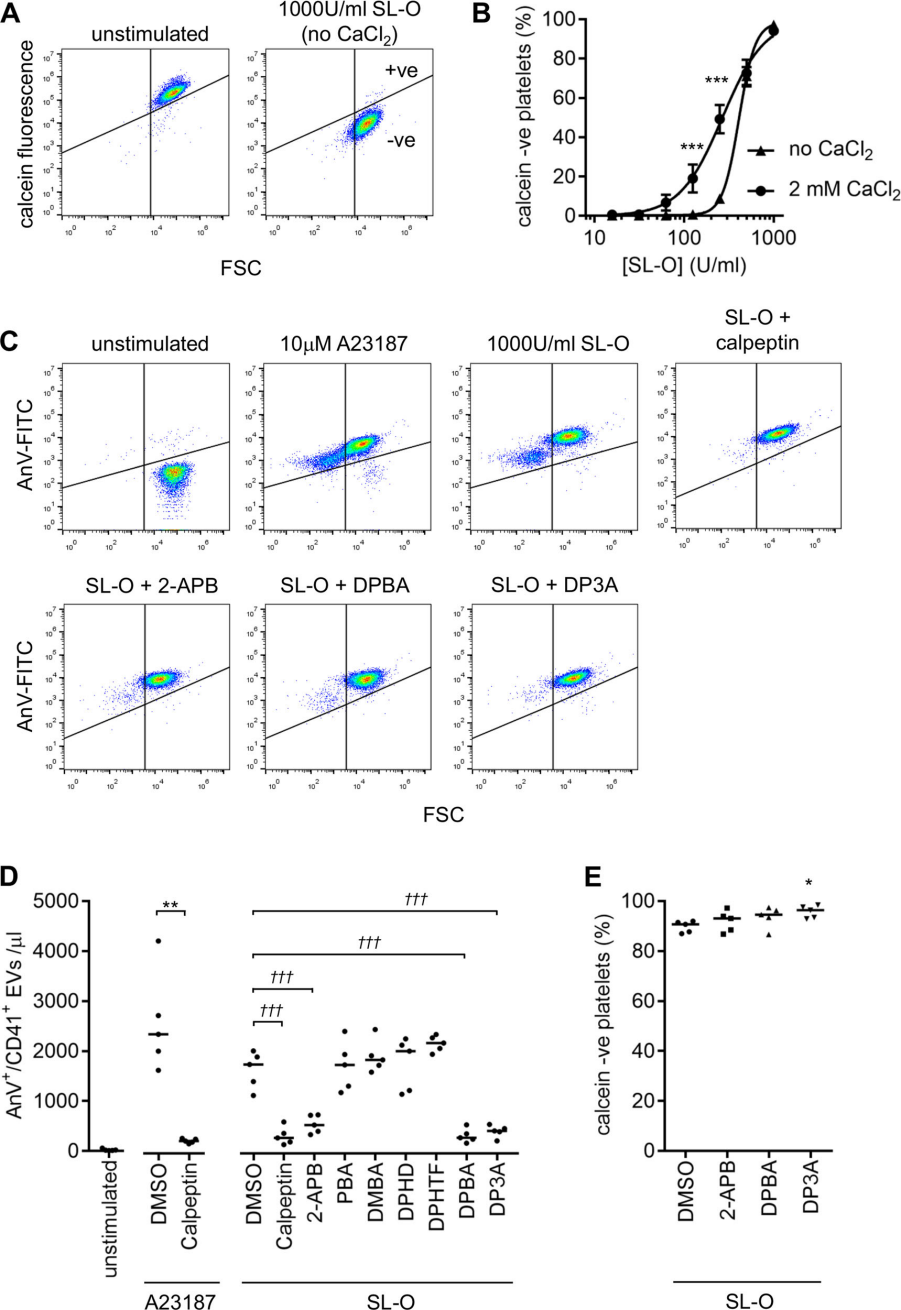
2-APB, DPBA and DP3A inhibit streptolysin–O-induced AnV^+^ release. **a**, **b** Platelets were loaded with calcein then treated with reduced streptolysin–O (SL–O) in the presence or absence of extracellular CaCl_2_. Representative density plots are shown in (**a**). Unstimulated platelets have high calcein fluorescence. SL–O treated platelets have lost calcein. **b** Mean ± s.e.m. (*n* = 5) of percentage of platelets that lost calcein fluorescence at various SL–O concentrations in the presence or absence of extracellular CaCl_2_. SL–O was more effective in the presence of extracellular CaCl_2_ (***p < 0.001; two-way RM-ANOVA with Sidak’s post-test). **c** Platelets (without calcein) were treated with SL–O then CaCl_2_ added. AnV^+^ EVs were released in a similar pattern to A23187-stimulated platelets. Where indicated, platelets had been pre-treated with calpeptin (140 µM; 30 min) or 2-APB, DPBA or DP3A (all 100 µM). Density plots are representative of five independent experiments. **d** AnV^+^ EV release from platelets stimulated with A23187 or SL–O, pre-treated as indicated. **p < 0.01 (calpeptin vs. DMSO, A23187 stimulated; paired *t* test); ^†††^*p* < 0.001 (treatments indicated vs. DMSO, SL–O-permeabilised platelets; one-way RM-ANOVA; other treatments were not significantly different *p* > 0.05). **e** Calcein-loaded platelets were treated with 2-APB, DPBA or DP3A, then permeabilised with SL–O. The percentage of platelets negative for calcein fluorescence in five independent experiments is shown. *p < 0.05 compared to DMSO (one-way RM-ANOVA with Dunnett’s post-test).

To determine whether the effect of 2-APB was due to directly interfering with the action of A23187, a different means of inducing AnV^+^ EV release was used. Platelets were permeabilised with the pore-forming toxin, streptolysin–O (SL–O). The efficiency of permeabilisation was tested by loading platelets with calcein. SL–O treatment led to loss of intracellular calcein. (Fig. 5a). Permeabilisation did not require extracellular Ca^2+^, though was enhanced by it (Fig. 5b). CaCl_2_ addition to permeabilised platelets triggered AnV^+^ EV release (Fig. 5c). AnV^+^ EV release was inhibited by the calpeptin (140 µM), indicating that calpain is required (Fig. 5c), which is similar to A23187-induced AnV^+^ EV release (Fig. 5d and ref. ^22^).

AnV^+^ EV release from permeabilised platelets was inhibited by 2-APB, DPBA and DP3A, but not the other analogues (Fig. 5c, d). This is the same pattern of action as seen with A23187-induced AnV^+^ EV release. None of the active drugs inhibited calcein loss from SL–O-permeabilised platelets (Fig. 5e)

### 2-APB and DPBA partially inhibits calpain activation, but DP3A does not

**Fig. 6:**
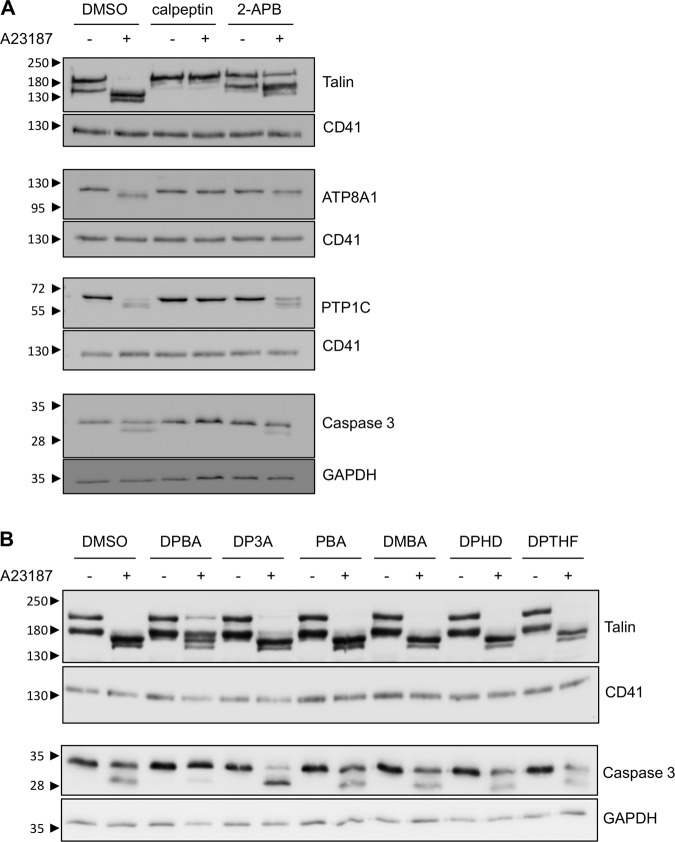
Calpain activity is inhibited by 2-APB and DPBA, but not DP3A. **a** Platelets were treated with the calpain inhibitor, calpeptin (140 µM), 2-APB (100 µM) or the vehicle (DMSO) prior to stimulation with A23187 as indicated, then lysed. Proteins were separated by SDS-PAGE. Talin, ATP8A1, PTP1C and caspase-3 were detected with specific antibodies. The membranes were then stripped and re-probed with anti-CD41-antibody or anti-GADPH antibody, as indicated. A23187-triggered protein cleavage was inhibited by calpeptin and partially inhibited by 2-APB. The blots are representative of five independent experiments. **b** Platelets were treated with the indicated analogues prior to stimulation with A23187. The blots are representative of five independent experiments.

Calpain is required for AnV^+^ EV release following A23187 or SL–O treatment. Platelet stimulation with A23187 resulted in cleavage of the calpain substrates, talin, ATP8A1, PTP1C and caspase-3, which was prevented by pre-treatment with calpeptin (Fig. 6a). For example, A23187 reduced the presence of the highest talin band to 8.5 ± 3.4% of unstimulated (DMSO-treated) samples (*n* = 5), whereas in the presence of calpeptin, this band was not significantly changed to 96.7 ± 6.1% of unstimulated, calpeptin-treated samples. Likewise, stimulation with A23187 led to the presence of a lower band (approximately 30 kDa) detected by the anti-caspase-3 antibody, consistent with previous reports^30^. However, in calpeptin-treated samples, stimulation of A23187 led to a barely detectable band, only 4.4 ± 1.0% (*n* = 5) of the band in DMSO-treated, A23187-stimulated samples. 2-APB (100 µM) also partially inhibited the cleavage of these substrates. In 2-APB-treated samples, A23187 reduced the highest band to 57.0 ± 11.0% compared to DMSO, unstimulated samples (*p* < 0.05; also *p* < 0.05 compared to DMSO, A23187-stimulated; one-way ANOVA with Tukey’s multiple comparisons test). Similarly, in 2-APB-treated samples, the lower caspase-3 band was only 40.8 ± 8.3% of that in DMSO-treated, A23187-stimulated samples (*p* < 0.05; *n* = 5). The 2-APB analogues were then tested for their ability to prevent the cleavage of two of these substrates, talin and caspase-3. Of the 2-APB analogues tested, DPBA also partially inhibited the cleavage of talin and capase-3, but DP3A, PBA, DMBA, DPHD and DPTHF had no effect (Fig. 6b).

### 2-APB also inhibits AnV^+^ EV release from endothelial cells

**Fig. 7:**
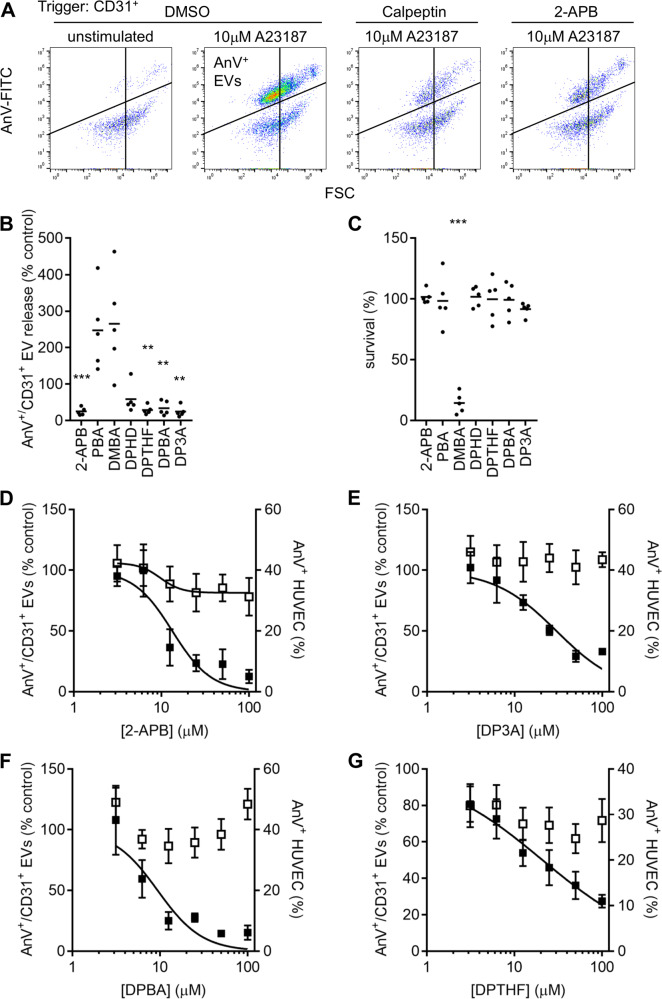
2-APB, DPBA and DP3A inhibit AnV^+^ EV release from HUVEC. **a** HUVEC were stimulated with A23187 (10 μM) as described in “Methods”. The panels show acquired events in the medium, stained with anti-CD31-APC and annexin V-FITC, using APC fluorescence to trigger acquisition. For comparison, some samples were pre-treated with calpeptin or 2-APB prior to stimulation. The density plots are representative of five independent experiments. **b** HUVEC were pretreated with 2-APB or one of its analogues then stimulated with A23187. AnV^+^ EV release into the medium in expressed as a percentage of AnV^+^ EV released in matched samples treated with the vehicle, DMSO, prior to A23187. **p < 0.01, ***p < 0.001 vs. DMSO; one-way ANOVA with Holm-Sidak’s post-test (*n* = 5). (**c**) HUVEC survival following treatment with 2-APB or its analogues was determined using a luminescence assay. ***p < 0.001. **d**–**g** Platelets were treated with a concentration range of the indicated analogue prior to stimulation with prior to stimulation with A23187 (10 µM). AnV^+^ EV release is expressed as a percentage of matched samples treated with the vehicle, DMSO, prior to A23187 (*n* = 5).

To determine whether the inhibitory effect of 2-APB was restricted to platelets, human umbilical vein endothelial cells (HUVECs) were stimulated with A23187 (10 μM), resulting in AnV^+^ binding to HUVECs and release of AnV^+^ EVs (Fig. 7a). AnV^+^ EV release was prevented by calpeptin, indicating that it is dependent on calpain activity (Fig. 7a). Pre-treatment with 2-APB (100 μM) inhibited the release of AnV^+^ EVs but not HUVEC AnV binding. The pIC_50_ for inhibition of AnV^+^ EV release was 5.88 ± 0.21 (Fig. 7d; *n* = 5), showing that 2-APB is more potent in HUVEC than platelets. The same range of 2-APB analogues was then tested (Fig. 7b, c). PBA unexpectedly increased AnV^+^ EV release. DMBA caused a significant loss of HUVEC viability, which may explain the increase in AnV^+^ EV release that it caused, and was not used further (Fig. 7c). AnV^+^ EV release was inhibited by DPBA (Fig. 7f; pIC_50_ = 6.03 ± 0.11; *n* = 5) and DP3A (Fig. 7e; pIC_50_ = 5.52 ± 0.32), as in platelets. However, in contrast to platelets, DPTHF also inhibited AnV^+^ EV release (Fig. 7g; pIC_50_ = 5.68 ± 0.20). DPHD also inhibited AnV^+^ EV release, but only at 100 μM. These data suggest that the pharmacology of AnV^+^ EV release from HUVEC is similar, but not identical, to that of platelets.

## Discussion

PS-exposing EVs released by activated platelets are prothrombotic and proinflammatory^11^, and so inhibiting their release may be a beneficial therapeutic approach^31^. However, little is known about the mechanisms that lead to their release and there are few drugs that can selectively inhibit this process. In this study, we demonstrate that 2-APB, a widely used Ca^2+^ channel modulator, inhibits PS-exposing EV release. This is not through inhibition of the major pathways currently known to be required for release of PS-exposing EVs and is not through inhibition of currently known targets of 2-APB. Although 2-APB itself is insufficiently selective or potent to be a useful therapeutic inhibitor of EV release, our results suggest the existence of a potential target. Future identification of this target may aid the development of therapeutic drugs that prevent the release of PS-exposing EVs.

Although little is known about the mechanisms of PS-exposing EV release from activated platelets, it requires a high, sustained increase in [Ca^2+^]_cyt_^32–34^. This can be triggered by a Ca^2+^ ionophore. Although this is not a physiological stimulation, it should bypass intracellular Ca^2+^ release channels, such as IP_3_Rs, and plasma membrane Ca^2+^ entry channels, such as Orai1 and TRPC6, major targets for 2-APB in platelets^24–26,35,36^. Indeed, this is why we were not expecting 2-APB to inhibit A23187-induced release of AnV^+^ EVs. In contrast, if physiological activators were used, such as thrombin plus cross-linked collagen-related peptide, the increase in [Ca^2+^]_cyt_ would require these Ca^2+^ channels^37,38^. The multiple effects of 2-APB mean that it would not itself be a suitable therapeutic, although understanding its mechanism of action may help us to understand the mechanisms that underlie the release of PS-exposing EVs from platelets.

Increased [Ca^2+^]_cyt_ activates the Ca^2+^-dependent scramblase, TMEM16F, leading to PS exposure, and activation of the protease, calpain. Both events are required for PS-exposing EV release^34^. It is not likely that 2-APB directly inhibits TMEM16F, as platelet AnV binding was largely unaffected under conditions where AnV^+^ EV release was completely inhibited. Moreover, although a small inhibition of platelet AnV binding was observed when 2-APB was present, this was fully reversed by washing the platelets, whereas the inhibition of AnV^+^ EV release was not so readily reversed.

Since 2-APB has many identified targets, we characterised a small set of related molecules that might have differing pharmacology. Two analogues, DPBA and DP3A, retained efficacy with similar potency to 2-APB. The other analogues showed no inhibitory action at 100 µM in platelets. These observations indicated that neither the secondary amine, nor the boron atom, are absolutely required for the inhibitory action. They also suggest that the effect of 2-APB is not through inhibition of store-operated Ca^2+^ entry (SOCE), since DPTHF and DMBA (and to a small extent DPHD) have been shown to inhibit SOCE^39^ but had no effect on AnV^+^ EV release. Interestingly, the pattern of active and inactive analogues was similar to that reported for interleukin (Il)-1β release from mouse macrophages^40^ (see Supplementary Table 1). In that study the effect was attributed to inhibition of the NLRP3 inflammasome. However, since Il-1β may be released in EVs^41^, inhibition of EV release could contribute to the inhibition of Il-1β release.

Moreover, these analogues provided a small toolkit to help explore the mechanism of action of 2-APB. AnV^+^ EV release showed the same pattern of sensitivity to 2-APB analogues whether the process was triggered by A23187 in intact platelets or by Ca^2+^ addition to SL–O-permeabilised platelets. This supports the assumption that the two stimuli were triggering the same process. It also indicates that the lack of action of PBA, DMBA, DPHD and DPTHF was not due to insufficient plasma membrane permeability.

2-APB is unlikely to inhibit AnV^+^ EV release through an action on Ca^2+^ channels. This is shown by several lines of evidence. The most direct evidence is the observation that 2-APB also inhibited AnV^+^ EV release from SL–O-permeabilised platelets. SL–O permeabilisation allowed calcein to completely leak from the platelets, and has been previously used to introduce antibodies into platelets^42^. Extracellular Ca^2+^ rapidly triggered AnV^+^ EV release from SL–O-permeabilised platelets in a calpain-dependent manner. Together, these data indicate that there is direct continuity between the extracellular and intracellular environment. 2-APB did not prevent calcein leakage from SL–O-permeabilised platelets, indicating that it did not affect this continuity. Therefore, it is unlikely that 2-APB inhibits AnV^+^ EV release by inhibiting Ca^2+^ flux between the extracellular and intracellular environment. Consistent with this conclusion, inhibition of Orai1 or TRPC6 (or NCX, which acts in reverse-mode downstream of TRPC6^37^) did not affect AnV^+^ EV release. Moreover, it is unlikely that other ion fluxes, such as Na^+^, K^+^ or Cl^−^ are required. For example, Ca^2+^-activated K^+^ channels have previously been proposed to regulate platelet PS exposure and subsequent AnV^+^ EV release^29^, and these channels may be inhibited by 2-APB^28^. However, not only is it unlikely that K^+^ flux plays an important role once the platelets have been permeabilised, but also two unrelated, non-selective blockers of Ca^2+^-activated K^+^ channels had no effect on A23187-induced AnV^+^ EV release. Therefore, it is likely that 2-APB has a target in AnV^+^ EV release that is separate to plasma membrane ion channels.

At first sight, our observation that 2-APB inhibits the A23187-induced increase in Cal-520 fluorescence appears to lead to the opposite conclusion, as it suggests that 2-APB inhibits the increase in [Ca^2+^]_cyt_. It is unclear whether this is a real inhibition of [Ca^2+^]_cyt_, or an artefactual effect on Cal-520 fluorescence. It is possible that 2-APB and DPBA affect the fluorescence spectrum of Cal-520 (in a manner that DP3A does not). We first considered whether 2-APB might induce cytosolic acidification, as it has been previously shown to rapidly acidify Jurkat T cells^43^, though this required action on (unidentified) membrane proteins and may be a cell-type specific effect. Many Ca^2+^-sensitive fluorescent dyes are acutely sensitive to pH and our data show that Cal-520 is also sensitive to pH. However, 2-APB did not affect intracellular pH, assessed by BCECF fluorescence, suggesting that cytosolic acidification does not explain the reduced Cal-520 fluorescence. In addition, treatments that did alter intracellular pH (extracellular addition of NH_4_Cl or HCl) did not affect AnV^+^ EV release. This indicates that 2-APB does not inhibit AnV^+^ EV release by altering intracellular pH.

**Fig. 8:**
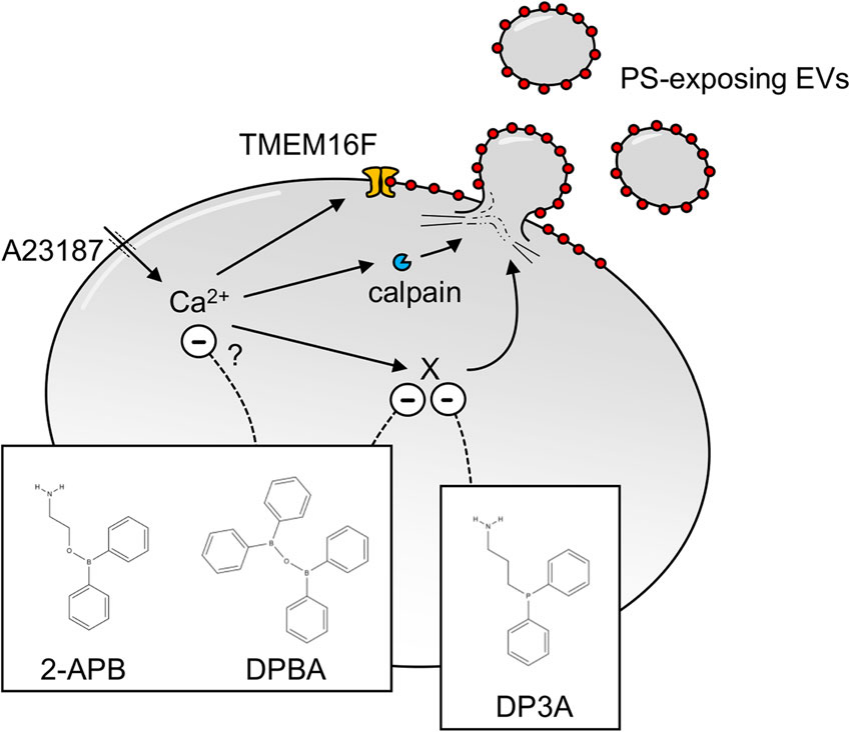
PS-exposing EV release is inhibited by 2-APB analogues. Summary of the targets of 2-APB, DPBA and DP3A. All three drugs inhibit PS-exposing EV release. 2-APB and DPBA also affect Cal-520 fluorescence, which may indicate inhibition of Ca^2+^ signalling, or may be artefactual (see “Discussion”). Inhibited Ca^2+^ signalling may explain the weak inhibition of Ca^2+^ effectors, TMEM16F and calpain, with 2-APB and DPBA. In contrast, DP3A does not show any inhibition of TMEM16F or calpain activity.

It is possible that 2-APB does inhibit the A23187-induced increase in [Ca^2+^]_cyt_, rather than artefactually decrease Cal-520 fluorescence, though the mechanisms that could be involved are unclear. The ionophore action of A23187 itself could be directly inhibited, which could be tested by using alternative approaches to increase [Ca^2+^]_cyt_ (e.g. ionomycin, thapsigargin). Our data indicate that the action of 2-APB on A23187-induced Cal-520 fluorescence does not involve inhibition of Orai1 or TRPC6, the major Ca^2+^ entry channels in platelets. One possibility is that platelets contain two intracellular Ca^2+^ stores, the dense tubular system and acidic Ca^2+^ stores, such as lysosomes^44^. The latter are relatively insensitive to Ca^2+^ ionophores^45^ and may require a 2-APB-sensitive process for their depletion. Inhibition of [Ca^2+^]_cyt_ signalling could account for the weak inhibition of PS exposure and calpain activity by 2-APB and DPBA. Although PS exposure and calpain activity are required for PS-exposing EV release^5,20,22,46^, these small inhibitory effects cannot account for the inhibition of AnV^+^ EV release by 2-APB, since the phosphorus-containing analogue, DP3A, had no effect on Cal-520 fluorescence, platelet AnV binding or calpain activity, yet inhibited AnV^+^ EV release with similar potency. These data suggest that there is a further target of 2-APB, independent of [Ca^2+^]_cyt_, PS exposure and calpain activity, that is required for AnV^+^ EV release (Fig. 8). DP3A is likely to inhibit the same target, without these other effects, and may be a better scaffold from which to develop a more selective inhibitor of EV release.

To determine whether the action of 2-APB was specific to AnV^+^ EV release from platelets, we also examined AnV^+^ EV release from HUVECs. Again, we used A23187 to directly increase [Ca^2+^]_cyt_, aiming to bypass any inhibition of Ca^2+^ channels by 2-APB. Endothelial cells release EVs during activation and apoptosis^47^. Whether the mechanisms underlying the release of EVs under these different conditions are the same in unclear. In our experimental setting, A23187 rapidly triggered AnV binding to HUVEC and release of AnV^+^ EVs. The latter was blocked by 2-APB. However, although AnV^+^ EV release from HUVEC was also inhibited by DPBA and DP3A, as in platelets, these drugs were more potent in HUVEC than in platelets. Moreover, AnV^+^ EV release from HUVEC was also inhibited by DPTHF. This suggests that the target of 2-APB in HUVEC and platelets may be similar but are not identical. As a tentative speculation, it may involve different isoforms of a protein family. This difference opens the possibility of being able to selectively inhibit PS-exposing EV release from different cells.

## Conclusion

PS-exposing EVs are released by stimulated platelets in a Ca^2+^/calpain-dependent manner. Although PS-exposing EVs are likely to contribute to a range of prothrombotic and proinflammatory diseases, there are few drugs available to block their release. 2-APB inhibits the release of AnV^+^ EVs. Identifying the target of 2-APB, DPBA and DP3A may provide a new way to inhibit AnV^+^ EV release from activated platelets and inhibit their contribution to thrombosis and inflammation.

## Methods

### Washed platelet preparation

Blood was drawn by venepuncture into sodium citrate (3.2% v/v) from healthy, drug-free volunteers, who had given written, informed consent in accordance with the Declaration of Helsinki. Use of human blood for these experiments was approved by the Human Biology Research Ethics Committee, University of Cambridge. Acid citrate dextrose (ACD; 85 mM tri-sodium citrate, 71 mM citric acid, 111 mM d-glucose) was added (1:7 v/v) and platelet-rich plasma (PRP) separated by centrifugation (200*g*, 10 min). Prostaglandin E_1_ (100 nM) and apyrase (Grade VII; 0.02 U/ml) were added to PRP to prevent platelet activation during preparation. Where required, platelets were incubated with either Cal-520-acetoxymethyl (AM) or calcein-AM (both 1 µM; 10 min). Platelets were pelleted from PRP by centrifugation (600*g*, 10 min) and resuspended in HEPES-buffered saline (in mM: 10 HEPES, 135 NaCl, 3 KCl, 0.34 NaH_2_PO_4_, 1 MgCl_2_·6H_2_O, pH 7.4; supplemented with 0.9 mg/ml d-glucose) at 5 × 10^7^ platelets/ml. Platelets were rested (30 °C, 30 min) prior to treatment with inhibitors or stimulation. CaCl_2_ (2 mM) was added immediately prior to simulation.

### Flow cytometry analysis

Following stimulation, samples were stained with FITC-conjugated annexin V (eBioscience, ThermoFisher, UK), to detect exposed PS, (FL1), unless otherwise indicated, and PE-Cy7-conjugated anti-CD41 antibody (eBioscience, ThermoFisher, UK), to distinguish platelet-derived events. Samples were analysed using a BD Accuri C6 flow cytometer. PE-Cy7 fluorescence (FL3) was used to trigger event acquisition. PS-positive platelet-derived EVs were defined as CD41^+^/annexin V^+^ events that were smaller than 1 µm. The 1 µm gate was set in forward scatter using 1 µm silica beads^22^.

### Immunoblotting

Platelet proteins were detected in platelet lysates by sodium dodecyl sulfate–polyacrylamide gel electrophoresis and immunoblotting, essentially as described previously^22^. The primary antibodies used were: anti-talin antibody (clone 8D4; T3287; from Sigma Aldrich, Poole, Dorset, UK) anti-PTP1C (610125; BD Biosciences); anti-ATP8A1 (21565-1-AP; Proteintech Europe, Manchester, UK); anti-CD41 antibody (ab134131; from Abcam, Cambridge, UK); and anti-caspase3 (9662) and anti-GAPDH (2118; from Cell Signalling Technology; Davers MA, USA). The secondary antibodies used were horseradish peroxidase-conjugated anti-rabbit IgG (7074) or anti-mouse IgG (7076; both Cell Signalling Technology).

### Measurement of cytosolic Ca^2+^ concentration ([Ca^2+^]_cyt_) and pH

Cal-520-loaded platelets were stimulated in black-walled microplates at 30 °C. Fluorescence (excitation: 492 nm; emission: 520 nm) was recorded using a FLUOStar OMEGA (BMG LabTech). BCECF-loaded platelets were stimulated in black-walled microplates at 30 °C. Fluorescence (excitation: 485 nm; emission: 520 nm).

### Measurement of endothelial EV release and PS exposure

HUVEC (Promocell, c-12203), were cultured in endothelial cell growth medium (PromoCell; c-22010), supplemented with 35 µg/mL gentamycin and endothelial cell growth supplements (Promocell, c-39215) at 5% CO_2_ and 37 °C. HUVEC were routinely tested for mycoplasma contamination. HUVEC were seeded into 24-well plates (at 1.5 × 10^5^/well) 24 h prior to drug treatment. HUVEC were washed to remove cell debris and constitutively released EVs then pre-treated for 30 min in endothelial cell growth media with the appropriate inhibitor. After this, media was replaced with HEPES-buffered saline supplemented with glucose (0.9 mg/ml), CaCl_2_ (2 mM), A23187 (10 μM) and inhibitor for 10 min. Following stimulation, both the EV-rich medium and trypsinized endothelial cells were collected and stained with APC-conjugated anti-CD31 (eBioscience,ThermoFisher, UK). Samples were analysed using a BD Accuri C6 flow cytometer. APC fluorescence (FL4) was used to trigger event acquisition and identify endothelial (or endothelial-derived EV) events. to determine EV count and cellular PS exposure, respectively. PS-exposing endothelial-derived EVs were defined as CD31^+^/annexin V^+^ events that were smaller than 1 µm in an analogous manner to platelet-derived EVs.

### Endothelial viability

To determine inhibitor toxicity, HUVEC were seeded in a 96-well clear-bottom plate at 1.5 × 10^4^ cells/well, 24 h prior to treatment. Endothelial cells were treated as described above. The cell viability assay (Promega, UK G7572) was conducted according to the manufacturer’s instruction to evaluate the ATP content.

### Data presentation and statistical analysis

Data are reported as mean ± standard error of mean (SEM) from at least five independent platelet preparations. *N* = 5 was chosen based on the mean and standard deviation of AnV^+^ EV release in preliminary experiments, in order to detect a 50% reduction with 80% power at *p* < 0.05. Data were compared using one-way or two-way repeat measures analysis of variance (RM-ANOVA), as appropriate, in GraphPad Prism v7. No samples were excluded from analysis. Concentration–response curves were fitted using a four-parameter logistical equation. This was also used to estimate pIC_50_ (i.e., −log(IC_5__0_)).

### Source of materials

All reagents, including the 2-APB analogues, were obtained from Sigma Aldrich (Poole, Dorset, UK) unless otherwise stated. A23187 was from Acros Organics (Fisher Scientific, UK). 2-APB was from Cayman Chemicals (Cambridge Bioscience, Cambridge, UK). SAR-7334 and SN-6 were from Tocris (Bristol, UK). Calcein and BCECF were from ThermoFisher Scientific. Cal-520 was from AAT Bioquest (Sunnyvale CA, USA).

## Supplementary information

Supplementary figures

## Data Availability

The data are available from the corresponding author on reasonable request.
