# Supplementary material for: 2-Aminoethoxydiphenylborate (2-APB) inhibits release of phosphatidylserine-exposing extracellular vesicles from platelets
Source: Cell Death Discov. 2020 Mar 2;6:10. doi: 10.1038/s41420-020-0244-9 (PMC7051957; doi:10.1038/s41420-020-0244-9)
Supplement: Supplementary file 1 — Supplementary figures [file 41420_2020_244_MOESM1_ESM.pdf]

Supplementary Table 1

Effect	2-APB	PBA	DPTHF	DPHD	DMBA	DPBA	DP3A	Ref.
This study								
AnV+ EV release	↓	-	-	-	-	↓	↓	n/a
AnV+ platelets	↓(weak)	-	-	-	-	↓(weak)	-	n/a
Cal-520 fluorescence	↓	-	-	-	-	↓	-	n/a
Calpain activity	↓	-	-	-	-	↓	-	n/a
Previous studies								
Thrombin-induced Ca^{2+} signalling (platelets)	↓	n.d.	↓	-	n.d.	↓	n.d.	[23]
SOCE inhibition	↓	-	↓	↓ (weak)	↓	↓	↓	[37]
SOCE potentiation	↑	-	-	-	-	↑	-	[37]
Il-1β release	↓	- (BC3)	-	-	↓ (weak) (BC12)	↓	n.d.	[38]

n.d. not determined
The compounds in Ref 36 are described by BCxxx, which refers to their designation in that study.

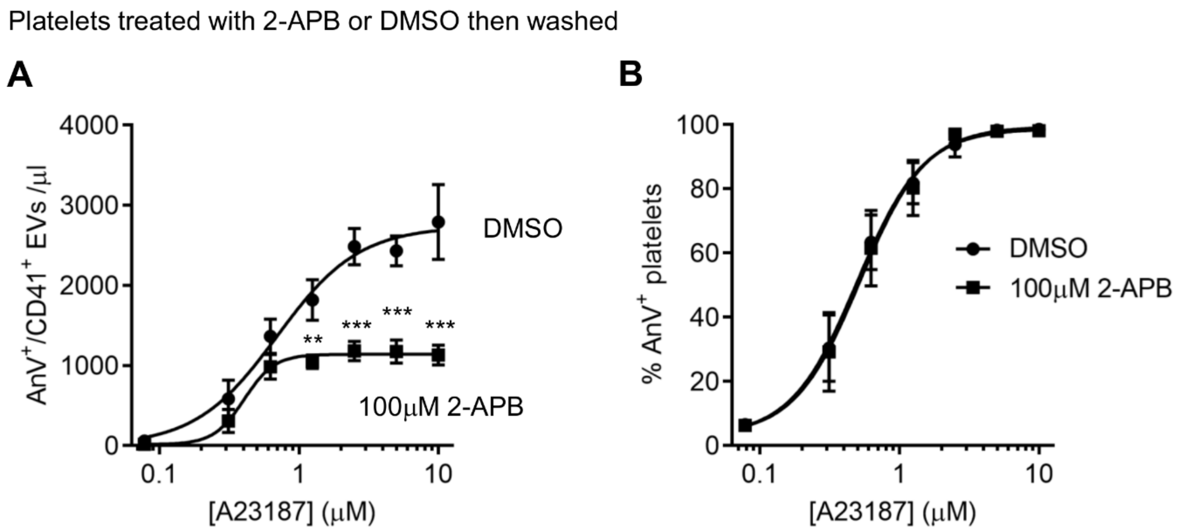

Supplementary Figure 1: Inhibition of AnV+ EV release by 2-APB is poorly reversible.

Washed platelets were treated with 2-APB (100 µM; 30 min) or DMSO. Platelets were then washed by centrifugation in the presence of apyrase and PGE_1. Resuspended platelets were stimulated with A23187 (10 µM). ** $p < 0.01$; *** $p < 0.001$ (n = 5; 2-way RM-ANOVA with Sidak's post-test.)

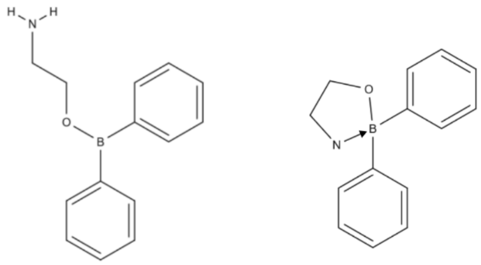

2-aminoethoxydiphenylborate (2-APB)

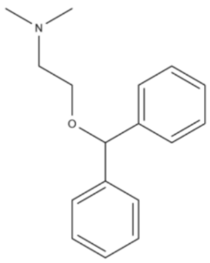

diphenhydramine (DPHD)

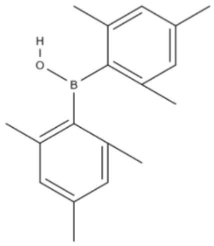

dimesitylborinic acid (DMBA)

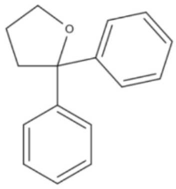

2,2-diphenyltetrahydrofuran (DPTHF)

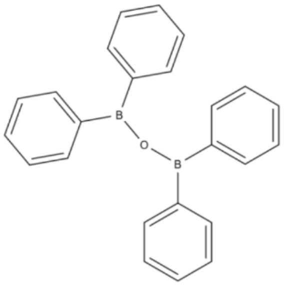

diphenylboronic anhydride (DPBA)

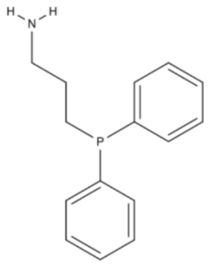

3-(diphenylphosphino)-1-propylamine (DP3A)

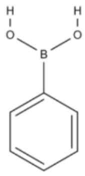

phenylborinic acid (PBA)

Drawn using http://molview.org/

Supplementary Figure 2: Structures of 2-APB and analogues used in this study.
2-APB can form a ring structure, which is mimicked by DPTHF.

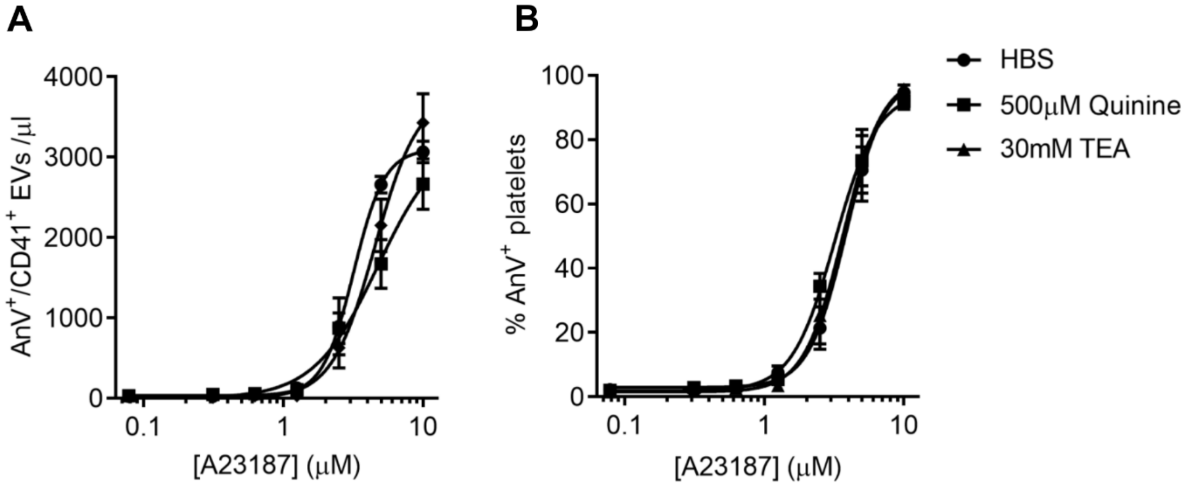

Supplementary Figure 3: Ca^{2+}-activated K^+ channels are not required for AnV^+ EV release.
Platelets were treated with quinine (500 µM) or TEA (30 mM) then stimulated with A23187. Data are mean ± s.e.m. (n = 5). No statistically significant difference was observed with either quinine or TEA compared to platelets treated with the solvent, HBS (2-way RM-ANOVA).
